# Targeting Cx43 and N-Cadherin, Which Are Abnormally Upregulated in Venous Leg Ulcers, Influences Migration, Adhesion and Activation of Rho GTPases

**DOI:** 10.1371/journal.pone.0037374

**Published:** 2012-05-15

**Authors:** Ariadna Mendoza-Naranjo, Peter Cormie, Antonio E. Serrano, Rebecca Hu, Shay O'Neill, Chiuhui Mary Wang, Christopher Thrasivoulou, Kieran T. Power, Alexis White, Thomas Serena, Anthony R. J. Phillips, David L. Becker

**Affiliations:** 1 Department of Cell and Developmental Biology, University College London, London, United Kingdom; 2 CoDa Therapeutics, Auckland, New Zealand; 3 Newbridge Medical Research Corp, Warren, Pennsylvania, United States of America; University Hospital Hamburg-Eppendorf, Germany

## Abstract

**Background:**

Venous leg ulcers can be very hard to heal and represent a significant medical need with no effective therapeutic treatment currently available.

**Principal Findings:**

In wound edge biopsies from human venous leg ulcers we found a striking upregulation of dermal N-cadherin, Zonula Occludens-1 and the gap junction protein Connexin43 (Cx43) compared to intact skin, and in stark contrast to the down-regulation of Cx43 expression seen in acute, healing wounds. We targeted the expression of these proteins in 3T3 fibroblasts to evaluate their role in venous leg ulcers healing. Knockdown of Cx43 and N-cadherin, but not Zonula Occludens-1, accelerated cell migration in a scratch wound-healing assay. Reducing Cx43 increased Golgi reorientation, whilst decreasing cell adhesion and proliferation. Furthermore, Connexin43 and N-cadherin knockdown led to profound effects on fibroblast cytoskeletal dynamics after scratch-wounding. The cells exhibited longer lamelipodial protrusions lacking the F-actin belt seen at the leading edge in wounded control cells. This phenotype was accompanied by augmented activation of Rac-1 and RhoA GTPases, as revealed by Förster Resonance Energy Transfer and pull down experiments.

**Conclusions:**

Cx43 and N-cadherin are potential therapeutic targets in the promotion of healing of venous leg ulcers, by acting at least in part through distinct contributions of cell adhesion, migration, proliferation and cytoskeletal dynamics.

## Introduction

Chronic wounds, such as diabetic foot ulcers, pressure ulcers, and venous leg ulcers (VLU) are an increasing problem worldwide, with estimates that 1–2% of the population in Western countries will develop a chronic wound over the course of their lifetime [1]. Chronic wounds represent a major economic burden on healthcare services with an estimated annual USA expenditure of $25 billion [2], [3]. With the growing numbers of elderly and diabetics in the population this expenditure figure is expected to rise in coming years. Unfortunately there is little in the way of effective therapeutic options for these debilitating wounds and there remains a significant need for effective new treatments.

Cx43 is the most ubiquitous connexin in the skin, expressed in keratinocytes and fibroblasts, endothelial cells and dermal appendages [4], [5]. We have reported that topical application of a Cx43-specific antisense containing gel to acute wounds in rodent models significantly accelerates the healing process whilst reducing inflammation and scar size [6], [7].

In the normal healing process Cx43 protein becomes down-regulated in keratinocytes in the first 24–48 hours as they become migratory and crawl forward to close the wound [8], [9], [10], [11], [12]. Following experiments in Cx43 conditional knockout mice it was later reported that downregulation of Cx43 appears to be a prerequisite for the coordinated proliferation and mobilization of keratinocytes during wound healing [13], [14]. In contrast, we showed that in STZ diabetic rats, a model for chronic wounds, Cx43 is upregulated in wound edge keratinocytes instead of being downregulated, and that migration is delayed until downregulation occurs [15]. Application of a Cx43 antisense to STZ diabetic rat wounds prevented the abnormal upregulation of Cx43 and restored wound closure to normal rates or better [15]. Over-expression of Cx43 was also shown to inhibit corneal endothelial wound healing in an *in vivo* rat corneal scrape injury model, while knockdown with Cx43 antisense sped it up [16]. Cx43 was also reported to be detected in the cells at the wound margins of the majority of biopsies taken from nine mixed and two diabetic leg ulcers [11].

One of the key impediments to the healing of chronic wounds is the failure of fibroblasts to migrate, proliferate and generate granulation tissue. Most previous reports have concentrated on epidermal Cx43 in wound healing and little attention has been paid to Cx43 in dermal fibroblasts. In the present work, we used a combination of *in vivo* and *in vitro* models to analyze the implications of elevated Cx43 expression, which we have discovered to be detrimentally upregulated in the dermis of human chronic VLU, and to correlate with reduced rates of migration of scratch-wounded fibroblasts over-expressing Cx43. In addition to Cx43, we also discovered that ZO-1 and N-cadherin, which interact with Cx43 and each other [17], are abnormally overexpressed in the dermis of human chronic VLU. Targeting Cx43 reduced the expression levels of ZO-1 and N-cadherin both *in vitro* and *in vivo*. Knock down of Cx43 and N-cadherin, but not ZO-1, accelerated cell migration in a scratch wound-healing assay. Reduction of Cx43 or N-cadherin also increased Golgi polarization whilst reducing proliferation and cell adhesion in fibroblasts. Targeting of Cx43 or N-cadherin, furthermore, was accompanied by cytoskeletal changes, increased lamellipodia protrusion, and activation of Rho GTPases. These findings support the idea that Cx43 and N-cadherin are therapeutic targets for improvement of wound repair through a mechanism involving remodeling of cell contacts and adhesion-dependent cytoskeletal modifications in fibroblasts.

## Results

### Cx43, N-cadherin and ZO-1 are dramatically upregulated in the dermis of human VLU

**Figure 1:**
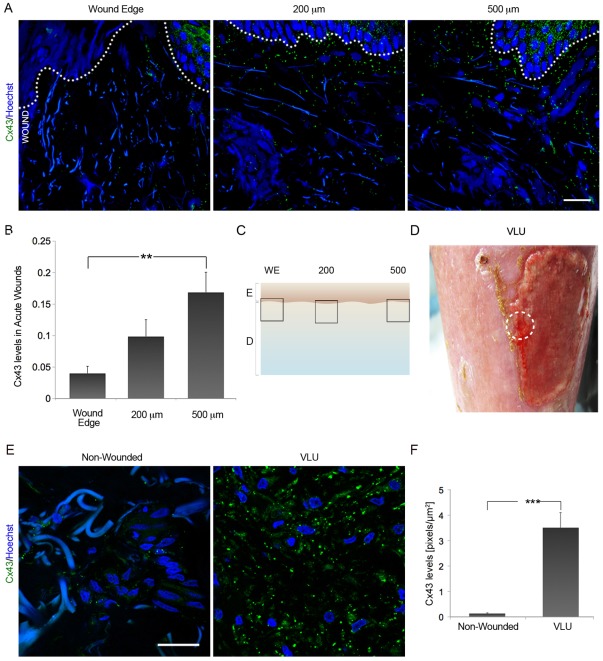
Dermal Cx43 is greatly upregulated in human chronic VLU. (A) Cx43 expression levels are reduced at the dermal wound margins 4 hours after leg punch biopsy of the skin of healthy volunteers. Scale bar = 25 µm. Blue signal is Hoechst staining of nuclei and collagen bundle autofluorescence. The dotted white line shows the border between the epidermis and the dermis (B) Cx43 levels increased with progressive distance from the injury site (C). Values represent mean ± SD (n = 3; **p<0.01). (D) A representative picture of a chronic VLU in the lower leg of a patient, from which a wound edge punch biopsy has been taken – white dotted circle. (E) Dermal Cx43 expression levels were significantly upregulated in chronic VLU in comparison to matched non-wounded samples (n = 6; *** *p*<0.005). (F) Graph depicting Cx43 levels in VLU vs. non-wounded skin. Values were expressed as mean ± SEM. Scale bar = 25 µm.

Skin punch biopsies, 2 mm, from healthy human volunteers (Figure 1A), and chronic wound edge biopsy samples, 4 mm, from 6 human VLU (Figure 1D and E), together with corresponding intact skin samples from the same patients (Figure 1E), were used to analyze Cx43 protein levels in the dermis of chronic vs. acute wounds. In acute wounds, downregulation of Cx43 was seen at dermal wound margins 4 hours after excisional wounding (Figure 1A), similar to that observed in the wound-edge dermis in a murine model after an excisional wounding (Figure S1, A) while Cx43 has been reported to persist at the wound edge epidermis of a majority of mixed diabetic leg ulcers [11]. Human and murine skin punch biopsies analysis showed that Cx43 was significantly reduced at the wound edge, with levels increasing towards normal with progressive distance from the injury site (Figure 1, A, B and C; *p*<0.01; and Figure S1, A, arrowheads; *p*<0.005). In sharp contrast, biopsies from the wound edge of VLU had not only failed to downregulate Cx43, but also showed significantly elevated expression of Cx43 throughout the dermis of the whole 4 mm biopsy (Figure 1, E and F; *p*<0.005).

**Figure 2:**
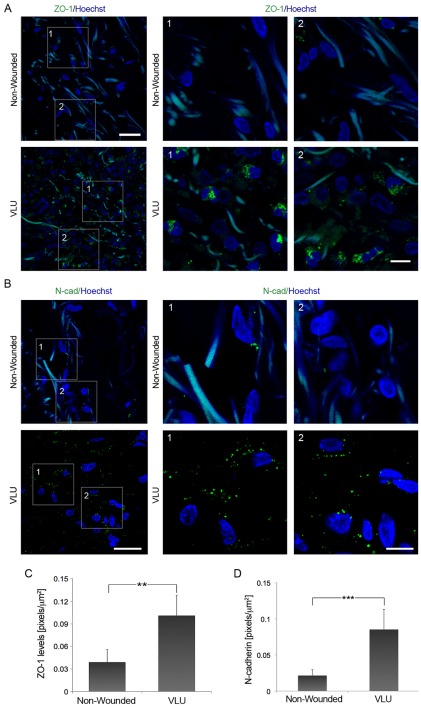
Increased expression of N-cadherin and ZO-1 in the dermis of human chronic VLU (A) ZO-1 expression levels are elevated in the dermis of chronic VLU compared to matched non-wounded controls (n = 6). Scale bar = 25 µm. Higher magnifications of VLU and intact skin (boxed regions 1 and 2) stained for ZO-1 (green) and Hoechst (blue) are shown. Scale bar = 10 µm. (B) N-cadherin is significantly upregulated in chronic VLU compared to matched non-wounded samples (n = 6). Scale bar = 25 µm. The boxed regions 1 and 2 show high magnifications of VLU and non-wounded skin samples stained for N-cadherin (green) and Hoechst (blue). Scale bar = 10 µm. Values represent mean ± SD. (C and D) Graphs show ZO-1 and N-cadherin expression levels in VLU vs. non-wounded skin. Values for ZO-1 and N-cadherin were expressed as mean ± SD; **p<0.01 and *p*<0.005, respectively.

Cx43 can interact with adherens junction proteins such as N-cadherin [17], [18], and with the tight junction associated protein ZO-1 [19] which also interact with each other. We investigated the expression and distribution of these proteins in the dermis of chronic VLU biopsies and matched non-wounded skin (Figure 2, A and B). ZO-1 and N-cadherin levels were significantly higher in chronic VLU samples than in matched non-wounded control dermis (Figure 2, C and D; *p*<0.01 and *p*<0.005, respectively). Taken together, these data indicate that human chronic VLUs not only fail to downregulate Cx43 in the dermal wound margins, but also that N-cadherin and ZO-1 are abnormally upregulated compared to their levels in intact skin.

### Cx43 expression is tightly linked to N-cadherin and ZO-1 expression in fibroblasts

We have previously shown that Cx43 downregulation significantly accelerates the skin healing process [6], [7]. Here we investigated the effects of Cx43 knockdown on ZO-1 and N-cadherin protein expression *in vivo*. To this end we used a mouse model of wound healing where full thickness skin excision wounds were treated with Cx43 antisense, or sense oligodeoxynucleotide control (Cx43asODN and Cx43sODN, respectively).

**Figure 3:**
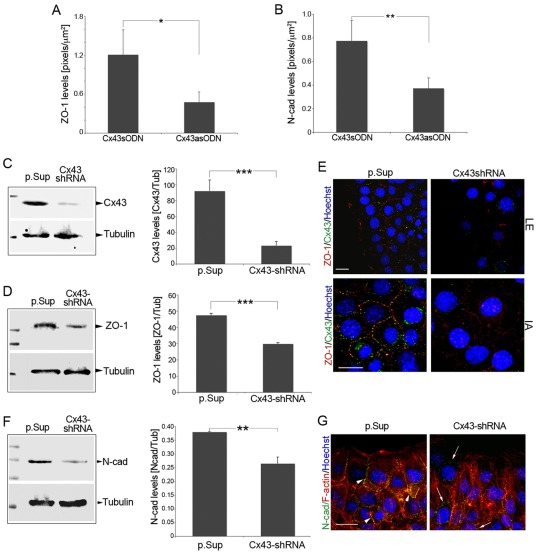
Targeting Cx43 reduces N-cadherin and ZO-1 expression in fibroblasts. (A) ZO-1 or (B) N-cadherin expression levels were examined in mouse skin wounds treated *in vivo* with Cx43sODN or Cx43asODN (n = 6). Downregulation of ZO-1 and N-cadherin was found in the dermis of mice after Cx43 knockdown. Graphs represent mean ± SD; * *p*<0.05 and **p<0.01. (C–D) ZO-1 and Cx43 levels were analyzed by Western blot in Cx43shRNA and p.Sup-transduced fibroblasts. Tubulin was included as loading control. Quantification shows that knockdown of Cx43 reduced the expression of ZO-1 (***p<0.005; n = 3). (E) ZO-1 (red) and Cx43 (green) expression and distribution in leading edge (LE) and internal areas (IA) were analyzed by confocal microscopy in wounded Cx43shRNA or p.Sup-transduced cells (n = 4). Cells were also counterstained with Hoechst (blue). Scale bar = 20 µm. (F) N-cadherin expression levels were significantly reduced after targeting Cx43 (**p<0.01; n = 3). (G) N-cadherin (green) redistribution from membrane (arrowheads) to cytosolic compartments (arrows) was analyzed along with F-Actin (red) in p.Sup and Cx43shRNA fibroblasts 3 h after scratch wounding of confluent 3T3 monolayers (n = 3). Scale bar = 20 µm.

Immunohistochemical analysis revealed positive ZO-1 staining in dermal sense-treated wounds, where ZO-1 was frequently co-localized with Cx43 (Figure S1, B). Treatments with Cx43asODN, on the other hand, induced a significant decline not only of Cx43, but also of ZO-1 in the dermal wound margins (Figure S1, B; Figure 3A, *p*<0.05). Analysis of N-cadherin expression revealed a preferential distribution in the dermis in sense-treated wounds, and significant protein downregulation after treatment with the Cx43asODN (Figure S1, C; Figure 3B, *p*<0.01). These data demonstrate that Cx43 expression is tightly linked to ZO-1 and N-cadherin expression in the dermis, and that targeting Cx43 can reduce ZO-1 and N-cadherin expression *in vivo*.

In order to better understand the effects of Cx43, N-cadherin and ZO-1 on fibroblast migration in response to a wounding stimulus we turned to the 3T3 fibroblast cell line. This can be easily transfected or transduced with shRNA constructs and avoids the variability of primary human fibroblasts derived from different patients. ZO-1 and N-cadherin expression was evaluated in fibroblasts transduced with a Cx43 short-hairpin RNA (Cx43shRNA) that suppresses Cx43 expression [20], or a p.Sup control plasmid. Cx43 protein expression and gap junction-mediated intercellular communication (GJIC) were effectively prevented in the Cx43shRNA cells, in contrast to p.Sup control cells (Figure 3C; Figure S2, G and I). With regard to ZO-1 expression, ZO-1 protein levels were downregulated in Cx43shRNA-transduced cells (Figure 3D). ZO-1 distribution was also analyzed in fibroblast scratch wound assays generated with Cx43shRNA and control cells. The Cx43shRNA-transduced cells showed a reduction of ZO-1 located at cell contacts at both the leading edge (LE) and in regions more internal to the wound (IA), accompanied by a loss of ZO-1 from the leading edge lamellipodia (Figure 3E, arrowheads). In p.Sup-transduced fibroblasts, however, ZO-1 was predominantly restricted to the contact margins between cells, often co-localizing with Cx43 (Figure 3E). With regard to N-cadherin, protein levels were significantly reduced in Cx43shRNA-transduced fibroblasts (Figure 3F; *p*<0.01) and N-cadherin was found located predominantly in the cytosol (Figure 3G, arrows) rather than the preferential plasma membrane localization found in p.Sup-transduced control fibroblasts (Figure 3G, arrowheads).

We also studied α- and β-catenin expression and distribution in p.Sup and Cx43shRNA-transduced cells. Whereas total β-catenin protein levels did not change significantly after Cx43 knockdown (Figure S3B), its cellular distribution was altered. In p.Sup-transduced cells, β-catenin was mainly localized to the plasma membrane at sites of cell-cell contact (Figure S3A), but relocation to the cytosol was additionally found in Cx43shRNA-transduced fibroblasts (Figure S3A, arrows), with evidence for some nuclear localization (Figure S3A, arrowhead). Alpha-catenin was seen localized in both the plasma membrane and the cytosol in p.Sup and Cx43shRNA-transduced cells (Figure S3C), and expression levels and location did not change with Cx43 knockdown (Figure S3D).

### Cx43 and N-cadherin, but not ZO-1 knockdown accelerate the rate of fibroblast migration

Targeting Cx43 for knockdown accelerates the rate of wound closure *in vivo* [6]. However, the underlying mechanism remains unknown. We investigated whether the increase in cell migration following a reduction in Cx43 expression was associated with reduction of ZO-1 protein levels or decreased cell adhesion (ZO-1 or N-cadherin down-regulation, respectively).

We first evaluated the migration rate of fibroblasts transduced with Cx43shRNA or p.Sup vectors, or cells treated with Cx43asODN or control Cx43sODN. Similar to Cx43shRNA cells, fibroblasts treated with Cx43asODN showed greatly reduced Cx43 protein levels and GJIC compared to Cx43sODN, or untreated cells (Figure S2, A and B, respectively; *p*<0.05 and *p*<0.01). We also transiently transfected fibroblasts with a bicistronic pIRES-GFP empty vector (pGFP) or with a matching vector encoding GFP and either wild-type Cx43 (Cx43-WT), or a dominant negative Cx43 construct (Cx43-DN) that interferes with the traffic of endogenous Cx43 protein to the plasma membrane, thereby blocking GJIC [21].

**Figure 4:**
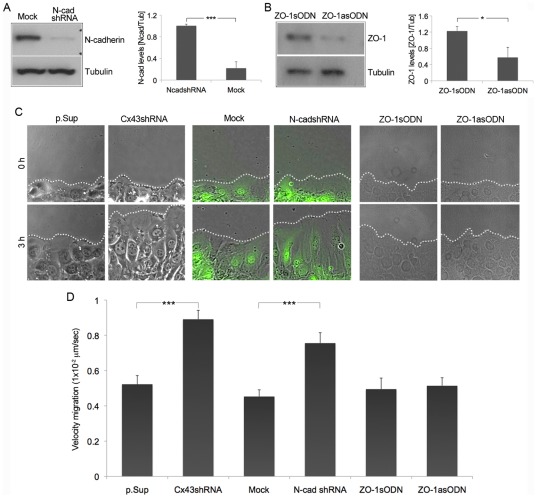
Cx43 and N-cadherin knockdown accelerates the rate of fibroblast migration. (A and B) N-cadherin and ZO-1 expression was evaluated by Western blot in Mock or N-cadshRNA-transduced cells, and in ZO-1asODN or sense-treated (ZO-1sODN) cells, respectively. Values represent mean ± SD (n = 3, ***p<0.005; n = 3, *p<0.05). (C) Cx43shRNA and p.Sup cells, Mock and N-cadshRNA cells, or fibroblasts treated with ZO-1sODN and ZO-1asODN were allowed to migrate into a wound for 3 h. Pictures of cells at the beginning of the migration recording (0 h) and at the end (3 h) are shown. (D) The graph shows the velocity of migration for all the aforementioned conditions. Values represent mean ± SEM (***p<0.005).

The velocity of migration of Cx43shRNA and Cx43asODN cells was significantly faster than p.Sup or Cx43sODN control cells, respectively (Figure 4, C and D, *p*<0.005; Movie S1; Figure S2, C and D; Movie S2). Cx43 knockdown cells also displayed much more extensive lamellipodia than control cells (Figure 4C, Movie S1). Similarly, Cx43-DN-transfected fibroblasts migrated faster than their untransfected neighbors or pGFP control cells, and also extended larger than usual lamellipodia at the leading edge (Figure S2, E and F; Movie S3). Conversely, fibroblasts overexpressing Cx43 (Cx43-WT) migrated significantly slower than either pGFP or Cx43-DN constructs (Figure S2, E and F; *p*<0.05 and *p*<0.01 respectively; Movie S3). These data provide further evidence for an inverse correlation between Cx43 protein levels and the rate of fibroblast migration after scratch wounding. However, there have also been reports that siRNA reduction of Cx43 can slow cell migration in 3T3 cell scratch wound assays that have been serum starved for 2 days [22]. We repeated these experiments in serum starved conditions and in the presence of normal amounts of cell culture serum. We found that in serum-starved conditions reducing Cx43 protein levels slowed migration whilst in more normal media conditions with serum, reducing Cx43 significantly sped migration (Figure S2 J).

We then analyzed the velocity of migration of fibroblasts transduced with N-cadherin or Mock shRNA constructs (N-cadshRNA and Mock, respectively). The N-cadshRNA construct effectively suppressed N-cadherin expression in NIH3T3 fibroblasts (Figure 4A), as previously described [23], which accelerated cell migration in scratch wound assays almost to the same extent as in Cx43shRNA transduced cells (Figure 4, C and D). To reduce ZO-1 expression in fibroblasts, cells were treated with ZO-1 sense and antisense oligodeoxynucleotide (ZO-1sODN and ZO-1asODN), previously described as able to effectively reduce ZO-1 expression [24]. Contrary to Cx43 and N-cadherin, ZO-1 down-regulation (Figure 4B) did not have any effect on the rate of fibroblast migration or wound closure (Figure 4, C and D). These results indicate that targeting Cx43 may additionally contribute to cell migration by reducing N-cadherin, but not ZO-1 protein levels in fibroblasts.

### Cx43 and N-cadherin regulate cell adhesion, polarization and proliferation in fibroblasts

**Figure 5:**
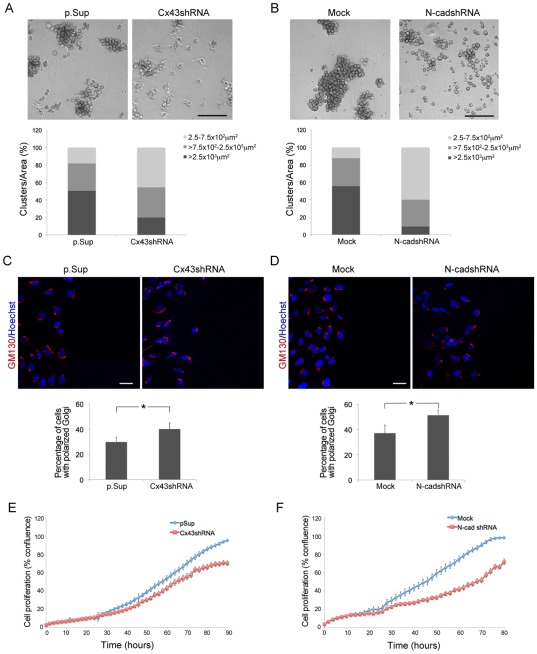
Cx43 and N-cadherin contribute to cell polarization, adhesion, and proliferation in fibroblasts. (A–B) Cell–cell adhesion upon suspension in hanging drops was assessed for Cx43shRNA and p.Sup, and for Mock and N-cadshRNA cells. The areas of cell clusters in six random fields taken from six different hanging drops were determined for each condition. Graphs represent the average number of clusters falling into each of three size ranges for each condition. Scale bar = 300 µm. (C–D) Cx43shRNA and p.Sup, and Mock and N-cadshRNA cells were wounded, allowed to migrate for 3 h, and then fixed and immunostained with anti-GM130 (red) and Hoechst (blue). Scale bar = 25 µm. The percentage of cells with Golgi located in the 120° arc facing the wound were scored as positive. Between 66–106 cells were evaluated in n = 3 independent experiments. Data represent the mean ± SEM (*P<0.05). Growth curves showing cell proliferation of fibroblasts transduced with (E) p.Sup and Cx43shRNA, and (F) Mock and N-cadshRNA constructs are shown. Data represent mean ± SD of three independent experiments, performed in duplicate.

Downregulation of the machinery of cell-cell adhesion is one of the ways in which different cell types, including skin cells, reactivate themselves to acquire a migratory phenotype [25]. Hanging drop assays have previously been used to characterize the strength of intercellular adhesion [26], [27]. We used this strategy to compare cell-cell adhesion properties by measuring the size of cell clusters that formed in suspended drops, and which resisted trituration with a micropipette tip [27]. Clusters were evaluated by morphometric image analysis and classified into three size groups according to their area [26]. Cx43shRNA and N-cadshRNA-transduced cells formed fewer trituration-resistant clusters (area >2.5×10^3^ µm^2^) and smaller clusters (area <7.5×10^2^ µm^2^) characteristic of reduced cell-cell adhesion (Figure 5, A and B; *p*<0.005).

The redistribution of the Golgi apparatus is an important event in the polarization and migration of many types of cells, including fibroblasts [28]. To examine the role of Cx43 and N-cadherin in cell polarity during cell migration, we analyzed the localization of the Golgi protein GM130 in fibroblasts transduced with Cx43shRNA, N-cadshRNA, or their respective controls. Increased GM130 polarization towards the wound was observed in Cx43shRNA and N-cadshRNA cells compared to p.Sup and Mock controls, 3 h after wounding (Figure 5, C and D; *p*<0.05).

Cell proliferation was also investigated using an IncuCyte™ live-cell imaging system. A clear reduction of cell growth was observed in Cx43shRNA and N-cadshRNA transduced cells, compared to p.Sup and Mock control cells (Figure 5, E and F), indicating a direct correlation between Cx43 and N-cadherin expression, and cell proliferation in fibroblasts.

Altogether these data confirm the important contribution of Cx43 and N-cadherin to the regulation of cell polarization, adhesion and proliferation processes in fibroblasts during wound repair.

### Targeting Cx43 induces cytoskeletal changes in leading-edge fibroblasts

**Figure 6:**
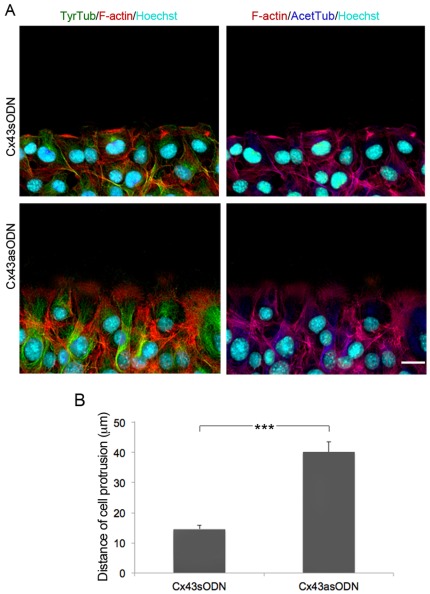
Targeting Cx43 induces cytoskeletal changes in leading-edge fibroblasts. (A) Representative images showing the distribution of F-actin (red), and tyrosinated (green) and acetylated (blue) tubulin (TyrTub and AcetTub, respectively) in leading edge Cx43sODN and C43asODN fibroblasts, 3 h after wounding. Cells were also counterstained with the nuclear marker Hoechst. Scale bar = 20 µm. (B) The graph shows the length of the protrusions of wound edge cells. Data represent the distance (mean ± SEM) from the nucleus to the leading edge in n = 3 experiments (***P<0.005).

**Figure 7:**
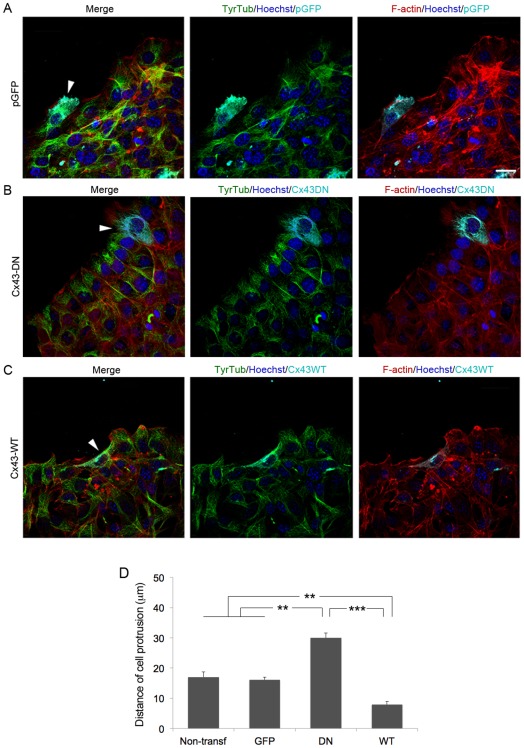
Modulation of Cx43 levels influence wound-edge cytoskeletal architecture in fibroblasts. Representative images of leading-edge cells transfected (arrowhead) with (A) a control pGFP construct (B) Cx43-DN, or (C) Cx43-WT, stained for TyrTub, F-actin and Hoechst. Scale bar = 20 µm. (D) Graph depicting the length of lamelipodial protrusions of fibroblasts transfected with the different constructs is shown (**p<0.01; ***P<0.005; n = 3).

Using scratch wound assays we observed that polarized Cx43 knockdown cells in the wound edge extended larger than usual lamellipodial protrusions (Movies S1 and S2). Directional cell locomotion requires complex interactions between actin filaments (F-actin) and microtubules, and we analyzed the distribution of F-actin, and tyrosinated and acetylated tubulin in wounded monolayers after targeting Cx43 expression in fibroblasts. Front-row control Cx43sODN, pGFP-transfected and p.Sup fibroblasts displayed the F-actin belt typical of polarized migratory cells (Figures 6A and 7A arrowhead; Figure S4A), which is regulated by adherens junctions [29]. In contrast, Cx43asODN, Cx43-DN and Cx43shRNA wound-edge cells developed rich lamelipodial protrusions oriented in the direction of movement, and lacked the F-actin belt found in control cells (Figures 6A and 7B arrowhead; Figure S4A). Under control conditions, tyrosinated and acetylated microtubules (TyrTub and AcetTub, respectively) were arranged very much as previously described in wounded fibroblast monolayer experiments [30], fanning out from the perinuclear region towards the wound margin. In Cx43asODN, Cx43shRNA, and Cx43-DN cells, which tended to be more extended, tyrosinated microtubules were predominantly oriented perpendicularly to the wound edge (Figures 6A and 7B arrowhead; Figure S4A). Cells over-expressing Cx43 (Cx43-WT) showed microtubules and lamellipodia that were less extensive than pGFP-transfected control cells (Figure 7C).

Lamellipodial dynamics were analyzed in more detail by transfecting fibroblasts with a red fluorescent protein (RFP)-actin construct. Sixteen hours after RFP-actin transfection a confluent monolayer was scratch-wounded and imaged for 1.5 hours by confocal microscopy. The p.Sup cells showed active, actin-rich lamellipodia and filopodia (Movie S4) and actin remodeling, characterized by dynamic formation and collapse of filopodia and actin ruffles in front-row cells. Cx43shRNA transduced fibroblasts displayed considerably more extensive actin-rich membrane protrusions at the front of the leading-edge cells (Movie 4). We quantified the extent of the cell protrusion and found that Cx43asODN, Cx43-DN and Cx43shRNA cells displayed almost twice the average protrusion length exhibited by Cx43sODN, pGFP and p.Sup control cells (Figures 6B and 7D; Figure S4B).

Following demonstration that Cx43 knockdown reduced N-cadherin expression, which also contributes to accelerate cell migration in fibroblasts (Figure 4D), we analyzed the distribution of polymerized actin in N-cadshRNA and Mock transduced cells 3 h after wound-scratch experiments. Similar to Cx43 knockdown fibroblasts, wound edge N-cadherin-targeted cells extended rich lamellipodial protrusions oriented towards the direction of cell migration (Figure S5A), and quantification showed more extensive lamellipodia protrusions compared to Mock control cells (Figure S5B).

### Targeting Cx43 and N-cadherin increases Rac1 and Rho-A GTPase activities in fibroblasts

**Figure 8:**
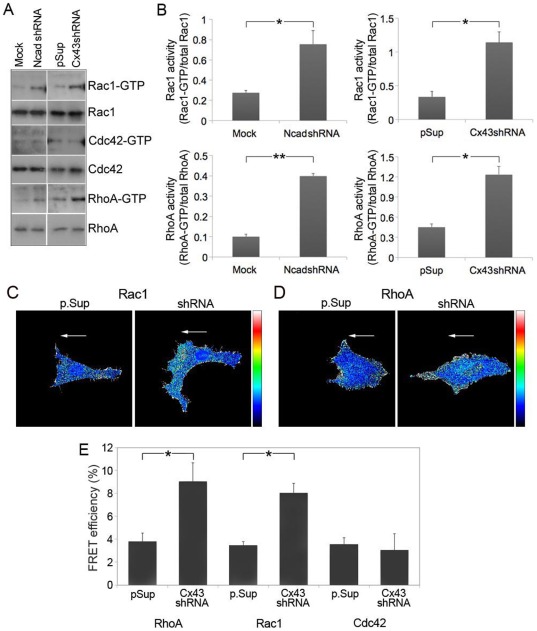
Cx43 and N-cadherin knockdown increases Rac1 and RhoA GTPase activity in fibroblasts. (A) Rho GTPase (GTP) activities in wounded fibroblasts were measured by pull-down assays using the PBD domain of PAK (Rac1 and Cdc42), or the RBD domain of Rhotekin (RhoA), followed by immunoblotting with the respective antibodies. Additionally, Rac1, Cdc42 and RhoA from total lysates were used as loading controls. (B) Graphs show active Rac1 and RhoA GTPase activity (GTP levels/total levels; mean ± SEM, *P<0.05); n = 3 independent experiments. Representative images of leading edge Cx43shRNA and p.Sup transduced cells showing (C) Rac1, and (D) RhoA GTPase activities, 3 h after wounding of confluent monolayers. Scale bar = 10 µm. Arrows indicate the direction of migration. (E) FRET efficiency analysis show a two-fold increase in Rac1 and RhoA activities in Cx43shRNA vs. p.Sup-transduced cells, while no differences were observed for Cdc42. Data is representative of n = 3 experiments per condition; *p<0.05.

Rho GTPases are key regulators of actin and microtubule dynamics and have an essential role in controlling different actin-based structures critical for cell motility and chemotactic responses [31]. We examined the effect of Cx43 and N-cadherin knockdown on Rho family GTPase activation in fibroblasts, using pull-down assays with specific GST fusion protein-binding domains for activated Rac1, Cdc42 and RhoA (GTP-bound forms). Quantitative analysis showed enhanced Rac1 and RhoA activity in Cx43shRNA and N-cadshRNA cells compared to p.Sup and Mock controls, respectively (Figure 8, A and B). In contrast, no differences in Cdc42 GTPase activity were detected.

To examine the activation of Rho GTPases in more detail, biosensors for Rac1, RhoA and Cdc42 [32], [33] were transfected into either p.Sup or Cx43shRNA-transduced cells and FRET was used to examine the activity of these GTPases in leading edge fibroblasts 3 hours after scratch-induced migration. Targeting Cx43 with Cx43shRNA induced a two-fold increase in Rac1 and RhoA activity over the p.Sup control (Figure 8, C and E; *p*<0.05), but had no effect on the activity of Cdc42 (Figure 8E). These findings support a direct role for Cx43 in the regulation of cytoskeletal dynamics in fibroblasts during cell migration.

## Discussion

Chronic wounds are a significant and growing global problem and place a great burden on both patients and healthcare system resources. While chronic wounds can occur anywhere on the body, most fall within the categories of VLUs, diabetic foot ulcers and pressure ulcers, which fail to progress through an organized, orderly and timely sequence of wound repair. Acceleration or even stimulation of wound closure is important for these chronic wounds, which are often infected and inflamed, making them painful and debilitating for the growing numbers of elderly and diabetic patients, and putting them at risk of lower limb amputation. Here we show that Cx43 is markedly upregulated in the dermis of human chronic VLU, similar to what was found in the wound edge epidermis of STZ diabetic rats, a feature that underlies impaired migration and healing [15]. We do not have ethical approval to biopsy and treat human VLU with Cx43 antisense and then resample these hard to heal wounds with additional biopsies. We therefore utilised a fibroblast cell line where we could reliably manipulate Cx43 protein expression and explore the effects on the dynamics of cell migration in a scratch wound healing assay. Whilst this is not as authoratative as in vivo human studies, they are useful for exploring the effect of Cx43 modulation on fibroblast behaviour. In these cell-culture experiments, fibroblast migration was impaired when Cx43 protein levels and GJIC were elevated after transfection with a Cx43-WT construct. These findings support a conclusion that the impaired healing observed in human chronic VLU wounds might be a consequence of the reduced rate of fibroblast migration caused by elevated levels of Cx43 protein.

However, elevated levels of Cx43 may not be the only factor that compromises healing in VLU. Cx43 reportedly forms a multiprotein complex or ‘nexus’ with ZO-1, α- and β-catenin, and N-cadherin [17], [18], [34], proteins that are likely to influence both cell adhesion and migration in wound healing. In fact, when compared to intact skin, we found that both ZO-1 and N-cadherin were significantly upregulated in the dermis of human chronic VLU, along with Cx43 upregulation. Clinically relevant is the observation that silencing Cx43 accelerated the velocity of fibroblast migration, supporting the idea that this process can be therapeutically controlled. Using *in vitro* models it was recently reported that connexin mimetic peptides also improve the migration rates of dermal fibroblasts [35], as well as keratinocyte and fibroblast migration in organotypic models and 2D cultures [36], which further reinforce our observations. These studies reported that levels of Cx43 protein were not changed by the peptide but phosphorylation of Cx43 was increased and cell adhesion decreased [35], [36]. We found that directly targeting Cx43 protein production additionally reduced N-cadherin and ZO-1 protein levels *in vivo*, with redistribution of both proteins from the plasma membrane to the cytosol. In the case of ZO-1, the protein was noticeably lost from the leading edge lamellipodia, which is normally a feature of fibroblast migration in scratch assay wound healing [37] and this may bring about changes in the distribution of the cytoskeletal components to which it binds.

The cytoplasmic tail of Cx43 is able to interact with the PDZ2 domain on ZO-1 but this interaction can be competed out by the addition of a mimetic peptide (ACT-1) to the last 9 amino acids of the Cx43 tail [38]. This peptide has been reported to stabilize Cx43 in the cell membrane forming larger gap junction plaques [38]. Applied to acute skin lesions this peptide is reported to accelerate wound healing whilst reducing scar formation, actions that are similar to those of the Cx43 antisense [39], [40], [41]. The mechanism behind the reported action of the peptide on wound healing is not at all clear at present. Interestingly, while knockdown of Cx43 and N-cadherin accelerated cell migration in a scratch wound-healing assay, targeting of ZO-1 failed to speed up fibroblast migration, indicating that ZO-1 down-regulation does not account for the faster migration that occurs when Cx43 is downregulated. It would be interesting in future studies to see if the ACT-1 peptide is able to accelerate fibroblast migration.

The literature regarding GJ communication and cell migration contains conflicting reports. Elevated connexin expression has been associated with reduced migration [42], [43], while other authors have reported the opposite effect [44], [45]. Using a different approach, *in vitro* studies performed on NIH 3T3 cells showed that the dynamic spreading movement, over an hour, of individual isolated and non-wounded cells appeared to be reduced when Cx43 was downregulated with siRNAs [17]. This discrepancy may reflect the different cell states (wounded vs. non-wounded) and different models used (cell migration into a scratch-wound vs. the spreading of non-wounded cells in sparse cultures). More recently the same group reported that Cx43 KO mouse embryonic fibroblasts migrated more slowly in scratch wound assays than those with Cx43 [22]. The reasons for these divergent observations are not clear but may be related to their serum starving of the cells for 48 hours prior to performing the scratch wound assay. We repeated the migration experiments with control 3T3 cells and 3T3 cells transduced with Cx43shRNA to reduce Cx43 expression. One batch was serum starved for 2 days, whilst the other was not. Cultures were then scratch wounded and imaged for 4 hours. We found that serum starved cells with reduced Cx43 migrated more slowly whereas reducing Cx43 in the absence of serum starvation speeded migration. As serum starvation would not be expected to match the conditions found in an acute wound in vivo, where we and others have shown that down regulating Cx43 speeds migration of fibroblasts and keratinocytes, we can only assume that it is the abnormal conditions of serum starvation that is triggering the anomalous response.

Cadherin-mediated cell–cell adhesion is reported to coordinate junction development with cell movement and cell polarization, and to maintain junction integrity by forming links with actin filaments [46]. Disruption of N-cadherin has been reported to increase the rate of Schwann cell migration on astrocytes by enhancing both the number of migrating cells, and the maximum migration distance [47]. Here we demonstrated that targeting Cx43, which consistently reduced N-cadherin expression, significantly diminished cell-cell adhesion, while increasing cell polarization in fibroblasts. This was further demonstrated by targeting N-cadherin, which provided similar results. It is also possible that decreased cell-cell adhesion is contributed to by a direct loss of Cx43–Cx43 connexon docking in Cx43-targeted fibroblasts. This was previously confirmed in cadherin null human squamous carcinoma cells [48], and also in other models in which expression of Cx43 increased cell adhesitivity [49], [50]. In the same way, interfering with Cx43 in individual cells of the 8–16 cell mouse embryo has previously been shown to reduce adhesion and produce decompaction of the targeted cell [51], whereas ZO-1 has recently been described to be essential for the compaction step [52]. Based on the findings described here we conclude that Cx43 down-regulation may prevent maturation of stable cell–cell adhesions by reducing N-cadherin expression and/or docking of connexons themselves.

We found that sustained reduction of the expression of either Cx43 or N-cadherin in 3T3 cells, by transduction with shRNA resulted in reduced cell proliferation. At first this observation does not seem to fit with previous reports from our group and others relating to Cx43 and cell proliferation. We have found that applying Cx43 antisense to an excisional wound on a mouse results in increased proliferation of fibroblasts and nascent keratinocytes but this was 1, 2 & 7 days after the treatment by which time the antisense was no longer preventing Cx43 protein production [7]. Pollock et al., 2011 [36] reported that the mimetic peptide GAP27 could in some cases enhance cell proliferation at the leading edge of a scratch wound assay of keratinocytes or fibroblasts. This effect was brought about without apparently reducing Cx43 protein levels and so the differences observed when Cx43 protein is significantly reduced may reflect the direct effect of the presence of the Cx43 protein on cell proliferation rather than the effect of Cx43 based gap junctional communication.

Trafficking of Cx43 hemichannels to cell–cell junctions takes place through a pathway that is dependent on microtubules [18], and it is well known that the cytoplasmic tail of Cx43 is able to interact with various components of the cytoskeleton, including microtubules and actin [18], [53], [54], [55], [56]. Here we show that front-row migrating fibroblasts lacking N-cadherin or Cx43 adopt an elongated phenotype with lamellipodia extended into the wound bed in the direction of migration. In contrast, actin organization in control-wounded fibroblasts was characterized by the F-actin belt typical of polarized migratory cells, known to be regulated by adherens junctions [29]. We also identified enhanced Rac1 and RhoA activities in Cx43 and N-cadherin-targeted fibroblasts, yet the molecular mechanism by which these proteins regulate Rho GTPase activation remains to be elucidated. However, these findings correlate with a reported role for RhoA in regulating fibroblast protrusion as an initiator of actin polymerization at the onset of the protrusion-retraction cycle [57]. Rac1, on the other hand, can influence the reinforcement and stabilization of newly expanded protrusions [57], which may help to explain the faster migration and more extensive lamellipodia observed in Cx43-targeted fibroblasts. The organization of the microtubule cytoskeleton was also investigated and found to be altered after reducing Cx43 levels, indicating that Cx43 not only interacts directly with microtubules, but may also affect microtubule dynamics during fibroblast migration. A finding that is in agreement with recent observations by Francis et al 2011showing altered microtubule dynamics in Cx43 KO mouse embryonic fibroblasts [22].

Overall, this study provides insight into why chronic VLUs that we have found to overexpress Cx43 in the dermis are slow to heal, and the cellular mechanisms as to how reducing Cx43 and N-cadherin expression accelerates fibroblast migration, pointing to a potential therapeutic solution to this debilitating problem. Our findings support the concept that the role of Cx43 is not simply to form GJ channels, but also to stabilize a nexus or multiprotein complex comprising N-cadherin, amongst others, that is required for cell-cell adhesion and adhesion-dependent actin dynamics, and must be broken down to facilitate efficient fibroblast migration. A large Phase 2 clinical trial is now underway using the Cx43asODN to reduce Cx43 expression in subjects with VLUs after an initial clinical trial showed significant improvement in complete healing.

## Materials and Methods

### Human chronic VLU and matched intact skin samples

Collection of wound edge punch biopsies from chronic venous ulcer leg wounds and normal arm biopsies was approved by the Western Institutional Review Board, Olympia, Washington State, USA. Punch biopsies from the thigh of healthy volunteers were under approval by the Northern X Regional Ethics Committee, Auckland, New Zealand. All biopsies were taken after written informed consent was obtained and the clinical research executed in accordance with the principles of the Declaration of Helsinki. Briefly for the VLUs a single 4 mm punch biopsy was taken from the wound edge under local anaesthesia in patients with clinically confirmed venous ulceration. A second 4 mm punch biopsy was taken from normal skin on the forearm in the same subjects. In all, biopsies from 6 subjects with clinically confirmed VLU (3 male and 3 female; range 38–79 years) were taken: median ulcer duration 6 months (range 1.5–36 months), and median size 10 cm^2^ (range 2–113 cm^2^). The VLU biopsies were initially fixed overnight with 4% paraformaldehyde (PFA), then transferred to 25% sucrose, and stored at 4°C until processing. For immunohistochemistry, tissue biopsies were embedded in OCT (BDH, UK). Samples were cryo-sectioned (10 µm) for immunohistochemistry analysis. For the normal (acute) wound biopsies an initial 6 mm punch biopsy was performed under local anesthetic on the anterolateral thigh of 3 healthy male volunteers (range 20–36 years) and then 4 hours later the punched wound site was excised with a larger 10 mm punch, and the resulting wound sutured closed. The 2 mm wide donut shaped biopsy was immediately imbedded in OCT snap frozen in liquid nitrogen and then stored at −80°C until being cryosectioned before analysis.

### Mouse and rat cutaneous wound-healing model and ODNs application

Male, 8-week-old ICR mice or Sprague-Dawley rats from UCL's Biological Services Unit were maintained according to UK Home Office animal regulations. Excisional lesions were performed as previously described [7]. Single topical applications of 10 µM unmodified Cx43asODN and control Cx43sODN (Sigma-Genosys) were delivered to each of two independent wounds in 30% Pluronic F-127 gel (Sigma). Two days after wounding, animals were humanely sacrificed and the wound tissue was harvested.

### Cell cultures and ODNs treatment

3T3 fibroblasts were grown in DMEM-GlutaMAX™-1 (Gibco, Invitrogen) supplemented with 10% FBS (Gibco, Invitrogen) in 5% CO_2_ at 37°C. For ODN treatments fibroblasts were washed with PBS to eliminate any traces of serum, and were incubated in serum free media for 2 hours with 20 µM Cx43asODN or Cx43sODN [6] (Sigma-Genosys); or with 10 µM ZO-1asODN or ZO-1sODNs [24] (Sigma-Genosys). Following this incubation, the medium with the ODNs was removed and replaced by 10% FBS in DMEM.

### Transfection; retroviral and lentiviral constructs and transduction

A retroviral pSuper vector containing the Cx43-specific shRNA target sequence GGTGTGGCTGTCAGTGCTC [20], kindly donated by W.H. Moolenaar and here designated Cx43shRNA, was used to establish a stable knockdown of Cx43 in 3T3 fibroblasts. A pSuppressor (p.Sup) retroviral vector (Imgenex Co) was used as control. The packaging cell line GP2–293 (Clontech) was transfected with the p.Sup and Cx43shRNA constructs as previously described [58]. Mock and N-cad shRNA constructs [23], kindly provided by S Arai and T Suda, were transfected into HEK 293T cells according to [59]. 3T3 fibroblasts were transduced with retrovirus or lentivirus for 2 days, and Cx43shRNA and N-cadshRNA and Mock-transduced cells were selected on the basis of resistance to 2 µg/ml puromycin or 500 µg/ml of geneticin (for p.Sup).

Fugene HD (Roche) was used to transfect 70% confluent fibroblasts with 1 µg/ml pGFP, Cx43-DN or Cx43-WT constructs ([21]) according to manufacturer's recommendation. Media was changed the following morning and 24 h later cells were used on different set of experiments.

### Immunohistochemistry of murine and human skin samples

Immunostaining was carried out on 6-µm cryostat sections of wounded rat and mouse skin, or 10-µm human chronic VLU or matched non-wounded skin, fixed in acetone at 4°C for 5 minutes. Primary antibodies (Abs) for Cx43 (diluted 1∶2000; Sigma, 6219), N-cadherin (diluted 1∶100; Abcam, ab18203) and ZO-1 (diluted 1∶100; Zymed Laboratories, 61–7300) were incubated for 1 hour at room temperature. Sections were washed with PBS and then incubated with the respective secondary Abs: swine anti-rabbit FITC-conjugated (DAKO) and goat anti-mouse Cy3-conjugated (Pierce). Secondary Abs incubation in the absence of primary Abs was used as negative control. Sections were then counterstained for 10 minutes with 5 µg/ml of the nuclear dye Hoechst 33342 (Sigma) and single optical sections were acquired on a Leica SP2 confocal microscope (Leica Microsystems, UK). All parameters during image acquisition were kept constant throughout each experiment to allow direct comparison of all of the 8 bit digital images. Immunostaining levels were quantified per unit area using a well-established pixel-counting method [15] using ImageJ software (NIH). Images were converted to binary images using an identical threshold. Objects greater than 2 pixels were counted in order to generate a readout of the number of positive pixels per unit area for comparison between conditions.

### Immunostaining

Confluent fibroblast monolayers were wounded and fixed 3 h later with 4% PFA for 10 minutes and permeabilized with 0.1% Triton X-100. Primary Abs for Cx43 (diluted 1∶2000; Sigma), ZO-1 (diluted 1∶100; Zymed Laboratories), N-cadherin (diluted 1∶100; Abcam), β-catenin (diluted 1∶200; ab6302 Abcam), α-catenin (diluted 1∶200; C2081 Sigma), tyrosinated tubulin (diluted 1∶400; YL1/2; Ab6160 Abcam) and acetylated tubulin (diluted 1∶200; T7451 Sigma) were used according to the manufacturer's recommendations. Incubation with the appropriate secondary Abs (Alexa 488 or 633; Molecular Probes) was followed by 10 minutes application of 5 µg/ml of the nuclear dye Hoechst 33342 (Sigma). Secondary Ab incubation in the absence of primary Ab was used as negative control. TRITC-phalloidin (Sigma) was included in some experiments for visualization of F-actin. Cells were imaged using a 63×, 1.25 NA objective on a Leica SP2 confocal microscope (Leica Microsystems, UK). At least three images, from 3–5 independent experiments were acquired. All acquisition parameters were kept constant throughout each experiment and staining was quantified based on pixel counts [15].

### Golgi reorientation measurements

Measurement of Golgi reorientation was performed as described previously [60]. Confluent fibroblasts were scratched and incubated for 3 h. After this time, cells were fixed with 4% PFA and stained with anti-GM130 (BD Biosciences) and Hoechst 33342 nuclear stain (Sigma). One hundred cells in ten randomly selected fields were evaluated for Golgi orientation.

### Cell proliferation

3T3 fibroblasts (1×10^4^ cells) transduced with pSup, Cx43shRNA, Mock and N-cadshRNA constructs were plated into 96-well plates and cell growth was monitored in real-time using the IncuCyte™ live-cell imaging system (Essen Instruments, Ann Arbor, MI). Experiments were performed at least three times in duplicate and data was expressed as percentage of confluence.

### Measurement of protrusion length

Confluent fibroblasts transduced with Cx43shRNA, p.Sup, N-cadshRNA and Mock constructs, or transfected with pGFP, Cx43-DN or Cx43-WT were wounded and fixed with 4% PFA 3 h later. The cells were then stained with Hoechst 33342 nuclear stain (Sigma). To quantify the protrusions' length, the distance from the nuclei to the leading edge of wound edge cells was measured. In each of three independent experiments, 3–5 transfected cells, or 30 transduced cells in randomly selected fields were analyzed to calculate the average length of these protrusions.

### Western blot

Cell pellets from harvested fibroblasts (Cx43shRNA- or p.Sup-transduced) were suspended at 4°C in ice-cold RIPA buffer plus protease inhibitors. Equal amounts of protein were resuspended in Laemmli 4× sample buffer, separated by 10% SDS-PAGE and visualized with primary Abs to Cx43 (diluted 1∶2000; Sigma), ZO-1 (diluted 1∶500; Zymed Laboratories), α-catenin (diluted 1∶200; Sigma), β-catenin β-catenin (diluted 1∶1000; Abcam) or N-cadherin (diluted 1∶500; Abcam). Abs against α-tubulin or actin (both from Sigma) were used as loading controls. Secondary antibodies were HRP-conjugated, and protein levels were visualized using an enhanced chemiluminescence (ECL) system (Amersham). The ratio protein/tubulin or/actin was determined by scanning and quantifying the bands, using ImageJ software (NIH).

### Dye-transfer assay

Dye-injection was carried out according to the method described previously [61]. Cells were impaled under visual control and filled with dye by iontophoresis. Communication was assessed 1 minute after cessation of iontophoresis. At least 10 injections were performed for each set of experimental conditions. The coupling was tested in cells: a) treated with Cx43sODN, Cx43asODN, or control untreated cells; b) transiently transfected with Cx43-DN, Cx43-WT or pGFP; and c) Cx43shRNA or p.Sup-transduced cells. Images of microinjected cells were acquired with a 40×0.8 NA objective on a Leica DMLFS microscope (Leica Microsystems, UK) using Volocity acquisition software (Improvision/Perkin Elmer).

### Cell migration assay

Confluent cultures of Cx43sODN, Cx43asODN, ZO-1asODN and ZO-1sODN-treated fibroblasts; or Cx43shRNA, p.Sup, N-cadshRNA and Mock-transduced cells; as well as Cx43-WT, Cx43-DN and pGFP-transfected fibroblasts were subjected to a mechanical scratch-wound. In some experiments, Cx43shRNA and p.Sup fibroblasts were serum starved (SS) or incubated in the presence of serum (FBS) for 48 h as described [22]. After this period of time, cells were wounded and incubated in DMEM supplemented with serum. Time-lapse images were taken soon after wounding at intervals of 5 minutes for 3–4 hours, and were acquired on an inverted Zeiss LSM AxioPlan 400 fluorescence microscope (Zeiss, UK), equipped with a Orca CCD camera (Hamamatsu, UK), using a 40×1.2NA objective with an incubation chamber at 37° and 5% CO_2_. Time-lapse images were captured using OpenLab image acquisition software (Improvision/Perkin Elmer, UK). The migration velocities of individual fibroblasts were quantified using Volocity4 analysis software (Improvision/Perkin Elmer) by measuring the distance between the initial and final positions of leading edge cells 3 hours after wounding. For each experimental condition, velocities were presented as mean ± SD (µm/sec).

### Rho GTPase activition assays

Fresh lysates were used for Rac1/Cdc42 and RhoA-pulldown assays, using the Rac/Cdc42-binding domain (p21-binding domain, PBD) of PAK, as previously described [62], or GST-Rhotekin (kind gift of P Rodriguez-Viciana) to detect relative amounts of RhoA-GTP. Bound protein (GTP-bound Rac1, Cdc42 or RhoA) levels were detected by western blot. Total Rac1, Cdc42 and RhoA from total cell lysates were also analyzed as loading controls.

### Förster resonance energy transfer (FRET)

Plasmids encoding FRET probes [Raichu-Rac1, Raichu-Cdc42 [32] and RhoA biosensor [33] were transfected into Cx43shRNA or p.Sup-transduced fibroblasts using Fugene HD (Roche). The FRET efficiency of the cells expressing the EYFP-ECFP fusion construct was measured using confocal fluorescence microscopy and acceptor photobleaching. Leading-edge transfected cells were imaged using an Olympus FluoView 1000 laser scanning confocal microscope and a 60×1.4NA oil objective (Olympus Microscopes, UK). The CFP and YFP channels were excited using the 440 nm and 515 nm lasers, respectively. The two emission channels were 460–510 nm for CFP and 520–620 nm for YFP. The gain for each channel was set to approximately 75% of dynamic range (12-bit, 4096 grey levels) and offsets set such that backgrounds were zero. Pre- and post-bleach CFP and YFP images were acquired and the FRET mode was used to collect images for each channel after acceptor bleaching with 10–12 scans of the 515 nm argon laser line at maximum power (to bleach YFP). The Olympus FluoView 1000 software was used to analyze donor and acceptor intensity values before (pre) and after (post) bleaching and values were then extracted from pixels falling inside the cell of interest, as well as an equal-sized bleached region outside the cell, and the mean ratio was determined for each region. The FRET efficiency ratio over the whole cell was calculated using the following formula: FRET efficiency = [ID _(post)_−ID _(pre)_]/ID _(post)_, where ID_pre_ and ID_post_ refer to the intensity of the donor (CFP) before (pre) and after (post) photobleaching of the acceptor (YFP).

### Hanging drop assay

Approximately 20,000 single cells were suspended in 35 µl drops of 10% FBS in DMEM from the lids of 24-well dishes for 4 hours. Water was placed at the bottom of each well to maintain humidity. The drops were pipetted five times up and down with 200 µl yellow tips, fixed with 4% paraformaldehyde, and aliquots were spread on coverslips. Images of six random fields from six individual samples for each condition studied (p.Sup and Cx43shRNA-transduced cells) were taken with a 4×0.1 NA objective on an inverted Axiovert Zeiss LSM microscope. An area of approximately 2.5×10^2^–7.5×10^2^ µm^2^ corresponds to a cluster of 1–3 cells and 2.5×10^3^ µm^2^ corresponds to about 10 cells. The area of each cell cluster was determined using a custom-written plug-in for ImageJ software (NIH). The area occupied by single cells was measured in parallel to estimate the amount of cells per unit area.

### Statistical analysis

Statistical differences were determined using Wilcoxon Matched-Pairs Signed-Ranks Test for paired data, or one-way analysis of variance (ANOVA) for data sets of multiple comparisons. A two-tailed Chi-squared test was used for the analysis of cell cluster sizes in the hanging drop assay of adhesion. All data are presented as the mean ± SD except where stated. Criterion levels for the individual tests are given in the Results.

## Supporting Information

Figure S1 **Cx43 is downregulated in fibroblasts after wounding.** (A) The expression and distribution of Cx43 in mouse's skin dermis was examined by immunohistochemistry 2 d after excisional wounding. Wound-edge Cx43 was reduced in dermal fibroblasts after such wounds. Arrowheads show how Cx43 becomes more prevalent with increasing distance from the wound edge. Scale bar = 25 µm. Cx43 levels were quantified along the wound site and were significantly lower at the wound edge; *p*<0.005. Values are expressed as mean ± SD. (B) ZO-1 (green) and Cx43 (red) were examined in mouse skin wounds treated *in vivo* with Cx43sODN or Cx43asODN (n = 6). Cells were also counterstained with Hoechst (blue). A clear downregulation of ZO-1 was found in the dermis of mice treated with the Cx43asODN. Scale bar = 100 µm. (B) Excisional wounds treated with Cx43asODN or control Cx43sODN were used to evaluate the distribution of N-cadherin (green) by immunohistochemistry. Scale bar = 100 µm.
(DOCX)

Figure S2 **Knockdown of Cx43 induces cytoskeletal changes.** (A) Cx43 expression levels and (B) cell-cell communication were evaluated in Cx43asODN or LiCl-treated 3T3 fibroblasts, and in untreated or sense-treated (Cx43-sODN) controls. Values are expressed as mean ± SD (*p<0.05; **p<0.01 and ***p<0.005; n = 4). (C) Confluent monolayers treated with Cx43sODN, Cx43asODN or LiCl were wounded and allowed to migrate for 3 hours. Pictures of cells at the beginning of the migration recording (0 hours) and at the end (3 hours) are shown. Scale bar = 25 µm. (D) The graph shows the velocity of migration, which was inversely correlated with both dye coupling and Cx43 expression. Values represent mean ± SEM (*p<0.01; ***p<0.05; n = 6). (E) Cells transfected with Cx43-DN, Cx43-WT or pGFP constructs (n = 3), as well as Cx43shRNA or p.Sup-infected cells (n = 6), were allowed to migrate into a wound for 3 hours. Images were taken at the beginning of the migration recording (0 hours; arrows pointing to Cx43-DN, Cx43-WT, or pGFP-transfected cells) and 3 hours later (arrowheads pointing to Cx43-DN, Cx43-WT or pGFP). Cx43shRNA and Cx43-DN cells accelerated migration, while Cx43-WT slowed it. Scale bar = 25 µm. (F) The graph shows the velocity of migration for each treatment and confirms that either silencing of Cx43 or transfection with Cx43-DN induce a significant increase in the rate of migration relative to the other conditions analyzed. Data are expressed as mean ± SD (*p<0.05; **p<0.01; n = 4). (G) Cx43 levels and dye coupling after LY microinjection were assessed in Cx43shRNA or p.Sup-infected 3T3 fibroblasts. Cx43shRNA was effective in downregulating Cx43 and eliminating communication with neighbouring cells. Values represent mean ± SEM for Cx43 levels, and mean ± SD for cell coupling (***p<0.005; n = 4). (J) Cx43shRNA and p.Sup cells were grown in the presence (FBS) or absence (SS) of serum and allowed to migrate into a wound for 4 h. The graph shows the velocity of migration; values represent mean ± SEM of n = 3 independent experiments (***p<0.005).
(DOCX)

Figure S3 **Analysis of α- and β-catenin expression and distribution after targeting Cx43.** (A and C) Protein distribution of α- and β-catenin was analyzed in p.Sup and Cx43shRNA-infected fibroblasts 3 h after wound scratch of confluent 3T3 monolayers. Arrows in (A) indicate cytoplasmic relocation of β-catenin from the plasma membrane to the cytosol in Cx43shRNA-infected cells. The single arrowhead indicates a site of putative nuclear localization of β-catenin in Cx43shRNA-infected fibroblasts. Scale bar = 25 µm. (B and D) The expression levels of these proteins were also evaluated by Western blot and values were normalized with respect to actin or tubulin (Tub).
(DOCX)

Figure S4 **Knockdown of Cx43 induces cytoskeletal changes.** The distribution of TyrTub, AcetTub, and F-actin was studied 3 h after wounding confluent monolayers of Cx43shRNA and p.Sup transduced fibroblasts. Scale bar = 25 µm. The graph shows the length of the protrusions of wound edge cells and data represent the distance from the nucleus to the leading edge (mean ± SEM; n = 3 experiments; ***P<0.005).
(DOCX)

Figure S5 **Knockdown of N-cadherin increases lamellipodial protrusions.** (A) The distribution of F-actin was studied 3 h after wounding confluent monolayers of Cx43shRNA and p.Sup transduced fibroblasts. Targeting N-cadherin induces cytoskeletal changes in wound edge cells. Scale bar = 25 µm. (B) The graph shows the length of the protrusions of control (Mock) and NcadshRNA wound edge cells. Data represent the distance from the nucleus to the leading edge (mean ± SEM; n = 3 experiments; ***P<0.005).
(DOCX)

Movie S1 **Cx43 expression influences the velocity of migration scratch wounded fibroblasts.** Time-lapse recordings of cells treated with Cx43sODN, Cx43asODN or LiCl were performed at intervals of 5 min for a period of 3 h. Cx43asODN-treated cells migrated almost twice as fast as Cx43sODN treated cells and displayed an extensive lamellipodial protrusion at the leading edge. Incubation with LiCl significantly reduced the rate of migration.
(MOV)

Movie S2 **Knockdown of Cx43 accelerates the rate of fibroblasts migration.** Fibroblasts stably transfected with Cx43shRNA or p.Sup were monitored for 3 h and time-lapse images were taken at 5 min intervals. Cells transfected with the Cx43shRNA migrated faster than their respective controls, further confirming the close relationship between Cx43 expression levels and the rate of migration in fibroblasts. Cx43shRNA-transfected cells also displayed the same phenotype observed in Cx43asODN treated fibroblasts, characterized by the formation of extensive lamellipodia during migration.
(MOV)

Movie S3 **Cx43-DN transfected cells migrate faster than Cx43-WT or eGFP-transfected cells after wounding.** Fibroblasts were transiently transfected with bicistronic pIRES-GFP expression vectors encoding Cx43-WT, Cx43-DN or eGFP alone. The migration rates were monitored for 3 h and time-lapse images were taken at 5 min intervals. Cells transfected with the Cx43-DN migrated faster than their respective controls, and also extended a large lamellipodial membrane protrusion at the leading edge in the direction of the migration.
(MOV)

Movie S4 **Actin dynamics is affected after silencing Cx43.** The effect of silencing of Cx43 on actin cytoskeleton dynamics was analyzed after sequentially transfecting p.Sup or Cx43shRNA-stable transfected fibroblasts with an RFP-actin construct. Confocal time-lapse images were taken at intervals of 2 min for 1 ½ h after a scratch wounding confluent cultures.
(MOV)
