# Supplementary figures and images for: Targeting Cx43 and N-Cadherin, Which Are Abnormally Upregulated in Venous Leg Ulcers, Influences Migration, Adhesion and Activation of Rho GTPases
Source: PLoS One. 2012 May 15;7(5):e37374. doi: 10.1371/journal.pone.0037374 (PMC3352877; doi:10.1371/journal.pone.0037374)

**Figure S1. Cx43 is downregulated in fibroblasts after wounding.**


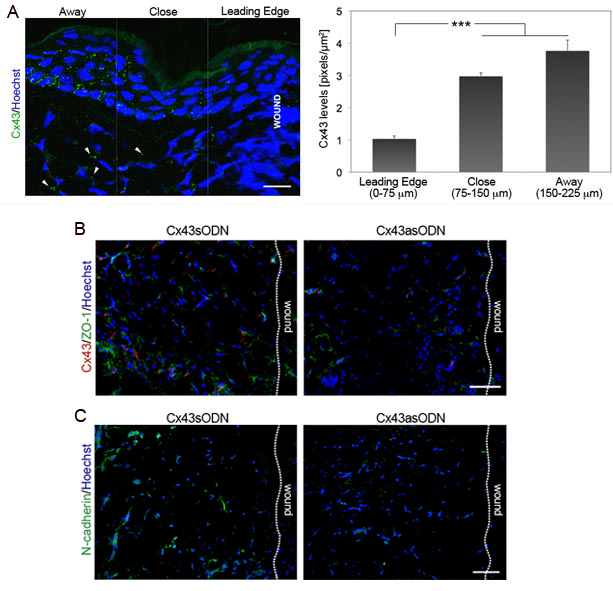

Supplement: Figure S1 — Cx43 is downregulated in fibroblasts after wounding. (A) The expression and distribution of Cx43 in mouse's skin dermis was examined by immunohistochemistry 2 d after excisional wounding. Wound-edge Cx43 was reduced in dermal fibroblasts after such wounds. Arrowheads show how Cx43 becomes more prevalent with increasing distance from the wound edge. Scale bar = 25 µm. Cx43 levels were quantified along the wound site and were significantly lower at the wound edge; p<0.005. Values are expressed as mean ± SD. (B) ZO-1 (green) and Cx43 (red) were examined in mouse skin wounds treated in vivo with Cx43sODN or Cx43asODN (n = 6). Cells were also counterstained with Hoechst (blue). A clear downregulation of ZO-1 was found in the dermis of mice treated with the Cx43asODN. Scale bar = 100 µm. (B) Excisional wounds treated with Cx43asODN or control Cx43sODN were used to evaluate the distribution of N-cadherin (green) by immunohistochemistry. Scale bar = 100 µm. (DOCX) [file pone.0037374.s001.docx]

**Figure S2 Knockdown of Cx43 induces cytoskeletal changes**


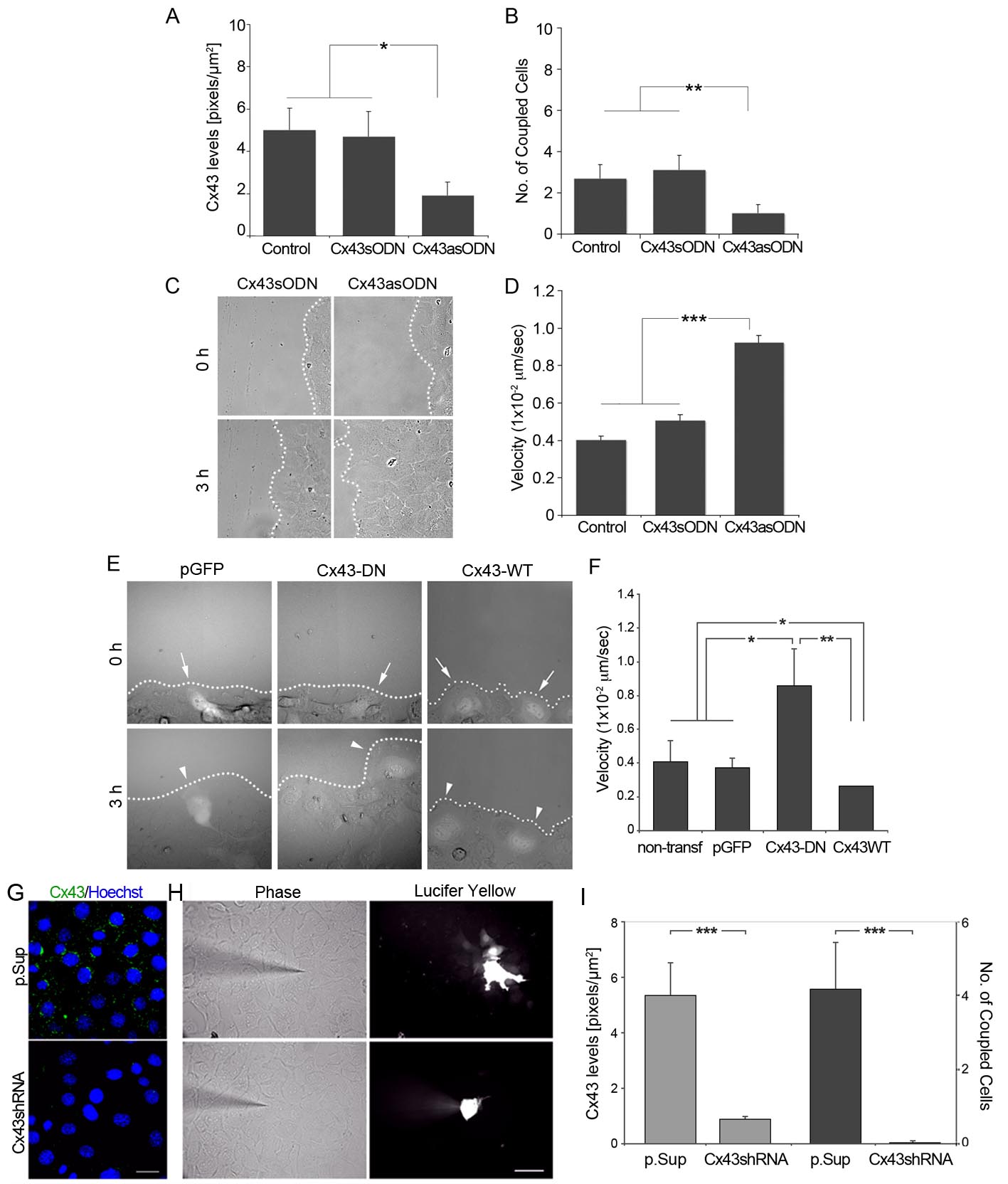

Supplement: Figure S2 — Knockdown of Cx43 induces cytoskeletal changes. (A) Cx43 expression levels and (B) cell-cell communication were evaluated in Cx43asODN or LiCl-treated 3T3 fibroblasts, and in untreated or sense-treated (Cx43-sODN) controls. Values are expressed as mean ± SD (*p<0.05; **p<0.01 and ***p<0.005; n = 4). (C) Confluent monolayers treated with Cx43sODN, Cx43asODN or LiCl were wounded and allowed to migrate for 3 hours. Pictures of cells at the beginning of the migration recording (0 hours) and at the end (3 hours) are shown. Scale bar = 25 µm. (D) The graph shows the velocity of migration, which was inversely correlated with both dye coupling and Cx43 expression. Values represent mean ± SEM (*p<0.01; ***p<0.05; n = 6). (E) Cells transfected with Cx43-DN, Cx43-WT or pGFP constructs (n = 3), as well as Cx43shRNA or p.Sup-infected cells (n = 6), were allowed to migrate into a wound for 3 hours. Images were taken at the beginning of the migration recording (0 hours; arrows pointing to Cx43-DN, Cx43-WT, or pGFP-transfected cells) and 3 hours later (arrowheads pointing to Cx43-DN, Cx43-WT or pGFP). Cx43shRNA and Cx43-DN cells accelerated migration, while Cx43-WT slowed it. Scale bar = 25 µm. (F) The graph shows the velocity of migration for each treatment and confirms that either silencing of Cx43 or transfection with Cx43-DN induce a significant increase in the rate of migration relative to the other conditions analyzed. Data are expressed as mean ± SD (*p<0.05; **p<0.01; n = 4). (G) Cx43 levels and dye coupling after LY microinjection were assessed in Cx43shRNA or p.Sup-infected 3T3 fibroblasts. Cx43shRNA was effective in downregulating Cx43 and eliminating communication with neighbouring cells. Values represent mean ± SEM for Cx43 levels, and mean ± SD for cell coupling (***p<0.005; n = 4). (J) Cx43shRNA and p.Sup cells were grown in the presence (FBS) or absence (SS) of serum and allowed to migrate into a wound for 4 h. The graph shows the velocity of migration; values repre [file pone.0037374.s002.docx]

**Figure S3 Analysis of α‑ and β‑catenin expression and distribution after targeting Cx43**.


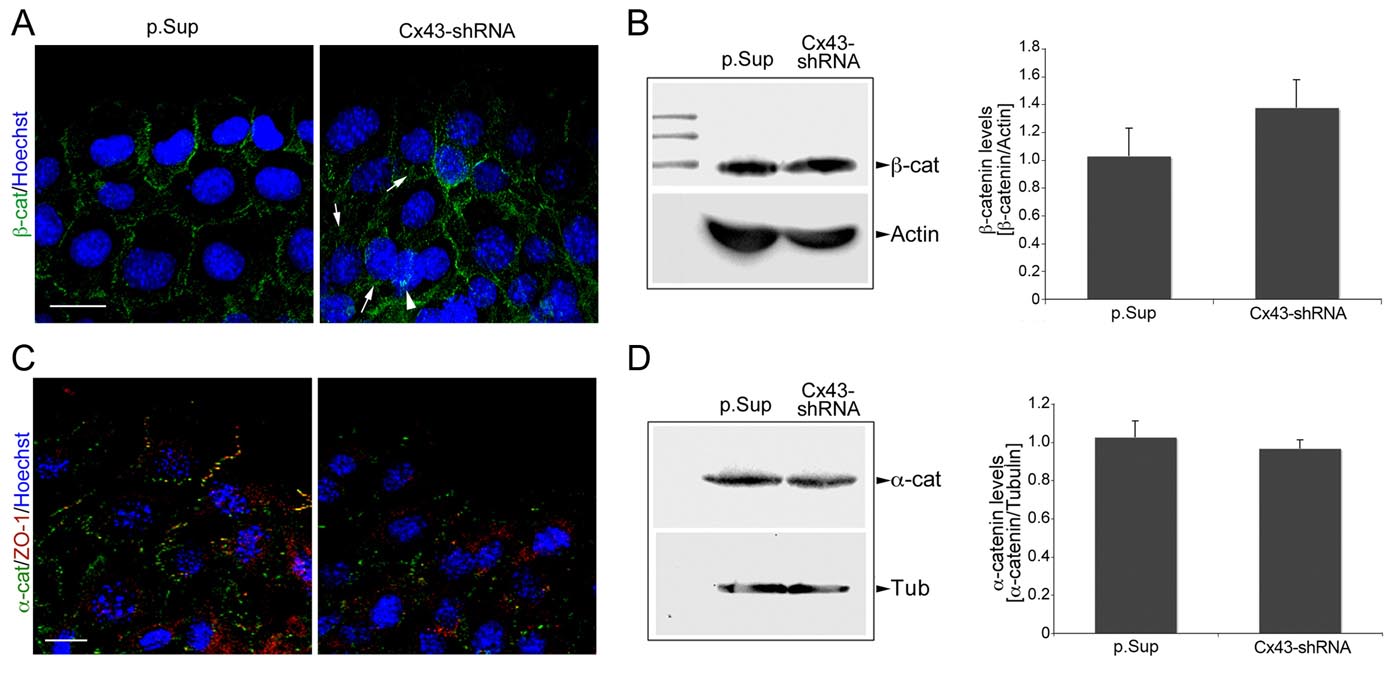

Supplement: Figure S3 — Analysis of α- and β-catenin expression and distribution after targeting Cx43. (A and C) Protein distribution of α- and β-catenin was analyzed in p.Sup and Cx43shRNA-infected fibroblasts 3 h after wound scratch of confluent 3T3 monolayers. Arrows in (A) indicate cytoplasmic relocation of β-catenin from the plasma membrane to the cytosol in Cx43shRNA-infected cells. The single arrowhead indicates a site of putative nuclear localization of β-catenin in Cx43shRNA-infected fibroblasts. Scale bar = 25 µm. (B and D) The expression levels of these proteins were also evaluated by Western blot and values were normalized with respect to actin or tubulin (Tub). (DOCX) [file pone.0037374.s003.docx]

**Figure S4 Knockdown of Cx43 induces cytoskeletal changes**.


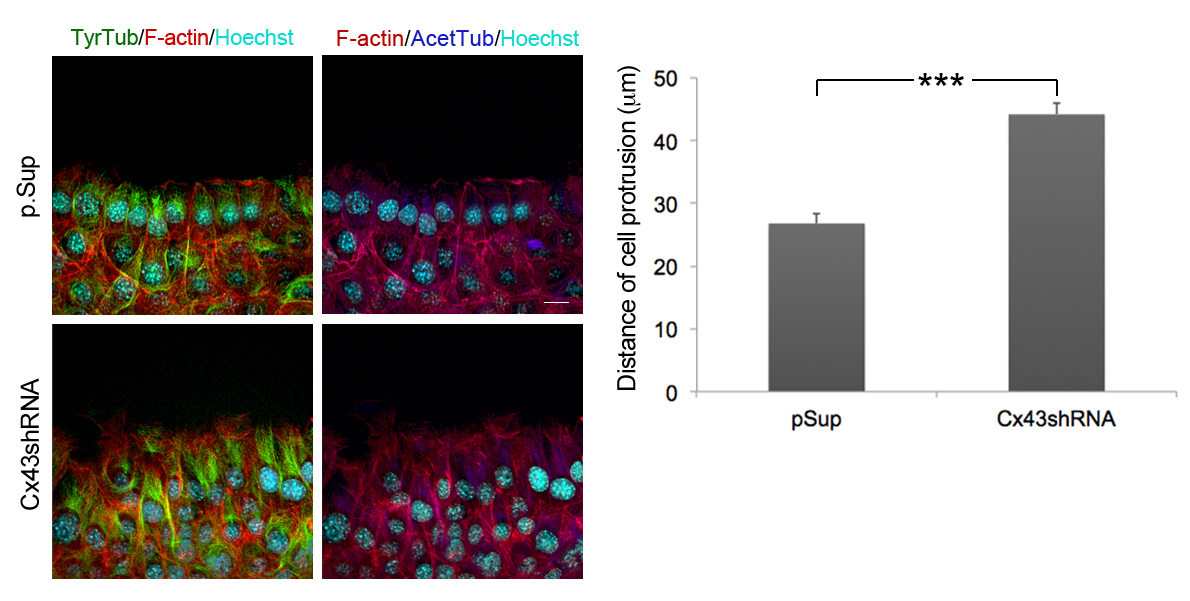

Supplement: Figure S4 — Knockdown of Cx43 induces cytoskeletal changes. The distribution of TyrTub, AcetTub, and F-actin was studied 3 h after wounding confluent monolayers of Cx43shRNA and p.Sup transduced fibroblasts. Scale bar = 25 µm. The graph shows the length of the protrusions of wound edge cells and data represent the distance from the nucleus to the leading edge (mean ± SEM; n = 3 experiments; ***P<0.005). (DOCX) [file pone.0037374.s004.docx]

**Figure S5 Knockdown of N-cadherin increases lamellipodial protrusions**.


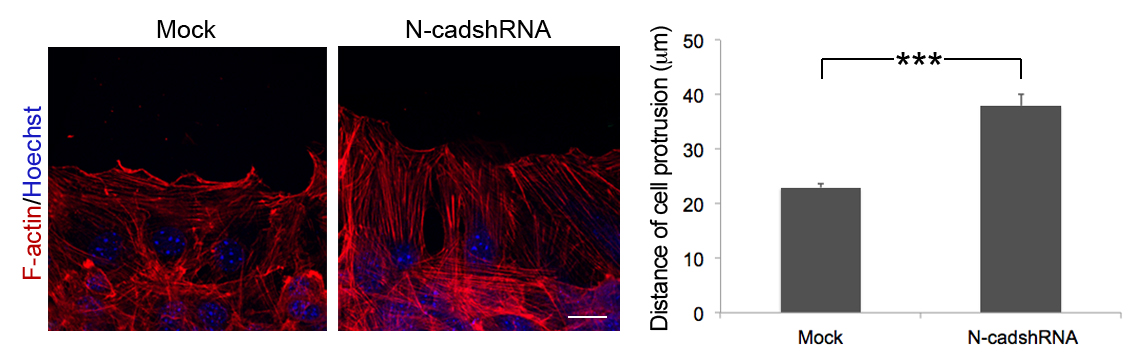

Supplement: Figure S5 — Knockdown of N-cadherin increases lamellipodial protrusions. (A) The distribution of F-actin was studied 3 h after wounding confluent monolayers of Cx43shRNA and p.Sup transduced fibroblasts. Targeting N-cadherin induces cytoskeletal changes in wound edge cells. Scale bar = 25 µm. (B) The graph shows the length of the protrusions of control (Mock) and NcadshRNA wound edge cells. Data represent the distance from the nucleus to the leading edge (mean ± SEM; n = 3 experiments; ***P<0.005). (DOCX) [file pone.0037374.s005.docx]
